# 2-(1,3-Di­thiol-2-yl­idene)-1,3-di­thiole-4-carbaldehyde

**DOI:** 10.1107/S160053681301711X

**Published:** 2013-06-26

**Authors:** Matthias Zeller, Vladimir A. Azov

**Affiliations:** a Youngstown State University, One University Plaza, Youngstown, OH 44555-3663, USA; b Department of Chemistry, University of Bremen, Leobener Strasse, NW 2C, D-28359 Bremen, Germany

## Abstract

The structure of the title compound, C_7_H_4_OS_4_, at 100 K has ortho­rhom­bic symmetry. In the crystal, tetra­thia­fulvalene mol­ecules form π-stacks along the *a* axis, with a stacking distance of 3.4736 (6) Å. Along the *b* axis, parallel stacks are inter­connected with each other through a network of weak C—H⋯O hydrogen bonds and short S⋯S contacts [3.4813 (7) Å]. Additional short S⋯S contacts [3.4980 (9) Å] join parallel stacks along the *c* axis.

## Related literature

For tetra­thia­fulvalene derivatives and their applications, see: Yamada & Sugimoto (2004 ▶); Segura & Martín (2001 ▶). For a review on synthetic chemistry of tetra­thia­fulvalenes, see: Fabre (2004 ▶). For a previous synthesis of the title compound, see: Garín *et al.* (1994 ▶). For reviews on ‘weak’ non-classical hydrogen bonding, see: Steiner (2002 ▶); Desiraju (2005 ▶). For reviews on halogen–halogen contacts, see: Metrangolo *et al.* (2008 ▶).
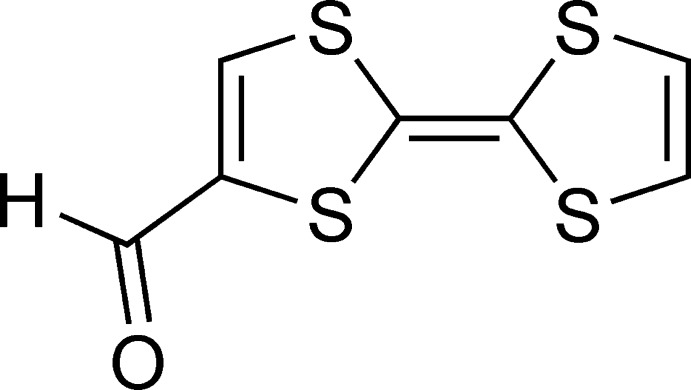


## Experimental

### 

#### Crystal data

C_7_H_4_OS_4_
*M* *_r_* = 232.34
Orthorhombic, 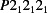

*a* = 3.8466 (3) Å
*b* = 7.4052 (7) Å
*c* = 30.577 (3) Å
*V* = 870.99 (13) Å^3^
*Z* = 4
Mo *K*α radiation
μ = 1.03 mm^−1^
*T* = 100 K
0.50 × 0.21 × 0.13 mm

#### Data collection

Bruker SMART APEX CCD diffractometer
Absorption correction: multi-scan (*SADABS* in *APEX2*; Bruker, 2012 ▶) *T*_min_ = 0.675, *T*_max_ = 0.746
6998 measured reflections
2734 independent reflections
2663 reflections with *I* > 2σ(*I*)
*R*_int_ = 0.015

#### Refinement

*R*[*F* ^2^ > 2σ(*F* ^2^)] = 0.024
*wR*(*F* ^2^) = 0.057
*S* = 1.13
2734 reflections
109 parameters
H-atom parameters constrained
Δρ_max_ = 0.45 e Å^−3^
Δρ_min_ = −0.25 e Å^−3^
Absolute structure: Flack *x* determined using 985 quotients [(I+)-(I-)]/[(I+)+(I-)] (Parsons & Flack, 2004 ▶), 1024 Friedel pairs
Flack parameter: 0.01 (4)

### 

Data collection: *APEX2* (Bruker, 2012 ▶); cell refinement: *SAINT* (Bruker, 2012 ▶); data reduction: *SAINT*; program(s) used to solve structure: *SHELXS97* (Sheldrick, 2008 ▶); program(s) used to refine structure: *SHELXL2013* (Sheldrick, 2013 ▶) and *SHELXLE* (Hübschle *et al.*, 2011 ▶); molecular graphics: *ORTEP-3 for Windows* (Farrugia, 2012 ▶) and *Mercury* (Macrae *et al.*, 2006 ▶); software used to prepare material for publication: *publCIF* (Westrip, 2010 ▶) and *enCIFer* (Allen *et al.*, 2004 ▶).

## Supplementary Material

Crystal structure: contains datablock(s) I, global. DOI: 10.1107/S160053681301711X/pk2488sup1.cif

Structure factors: contains datablock(s) I. DOI: 10.1107/S160053681301711X/pk2488Isup2.hkl

Supplementary material file. DOI: 10.1107/S160053681301711X/pk2488Isup3.cml

Additional supplementary materials:  crystallographic information; 3D view; checkCIF report

## Figures and Tables

**Table 1:** Hydrogen-bond geometry (Å, °)

*D*—H⋯*A*	*D*—H	H⋯*A*	*D*⋯*A*	*D*—H⋯*A*
C1—H1⋯O1^i^	0.95	2.38	3.228 (3)	149
C3—H3⋯O1^i^	0.95	2.69	3.445 (3)	137

Symmetry code: (i) 
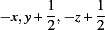
.

**Fig. 1.**
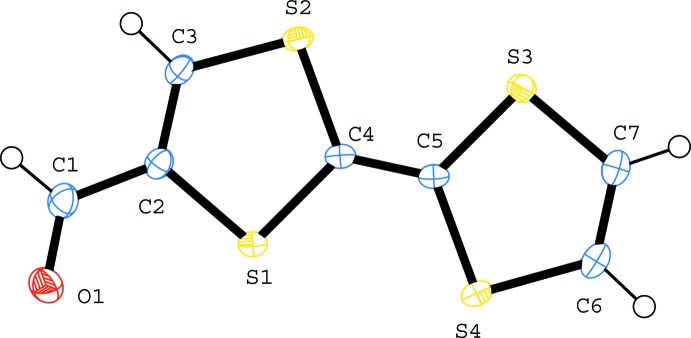
ORTEP–3 plot of the title molecule with the atom numbering scheme. Displacement ellipsoids are represented at 50% probability levels. H atoms are presented as a small spheres of arbitrary radius.

**Fig. 2.**
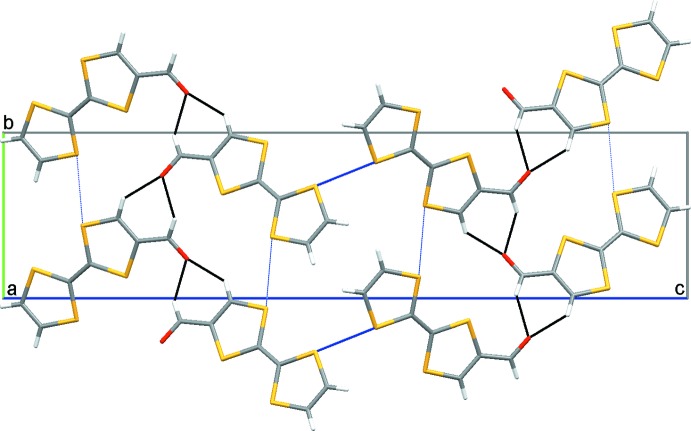
Crystal packing of the title compound viewed along the a axis. Hydrogen bonds are shown as solid black lines, short S3···S3 contacts are shown as solid blue lines, and short S2···S4 contacts are represented as dotted blue lines.

**Fig. 3.**
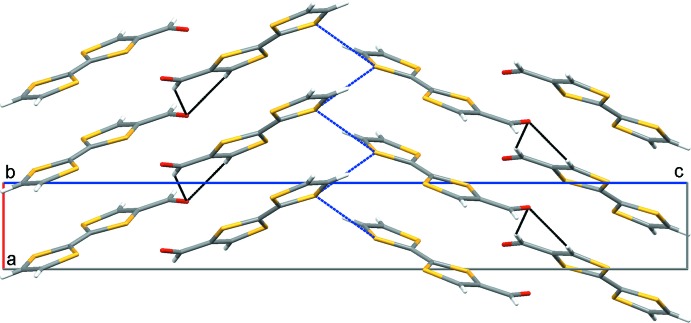
Crystal packing of the title compound viewed along the b axis. Hydrogen bonds are shown as solid black lines, short S3···S3 contacts are shown as solid blue lines.

## supplementary crystallographic information

### Comment

The title compound, commonly known as 4-formyltetrathiafulvalene, was prepared from tetrathiafulvalene (TTF) and can serve as an intermediate for the synthesis of 4-(hydroxymethyl)tetrathiafulvalene by reduction with NaBH_4_, of conjugated TTF derivatives by means of Wittig reaction (Garín *et al.*, 1994), of TTF imines by reaction with amines, and of other functional tetrathiafulvalenes (Yamada & Sugimoto, 2004; Fabre, 2004).

The molecular structure of the title compound with atom numbering scheme is shown in Fig. 1. Bond lengths and angles may be considered normal. The molecular framework excluding the carbonyl group is essentially planar, with a maximum deviation of fitted atoms from the least-square plane, defined by the heavy atoms of the TTF backbone, of 0.042 (2) Å for C6. Atoms of the carbonyl group show more substantial out of plane deviation of 0.128 (2) Å for C1 and of 0.2393 (19) Å for O1, respectively.

Details of the packing interactions are given in the Tables. Molecules of the 4-formyltetrathiafulvalene form π-stacks along the *a* axis with a distance of 3.4736 (6) Å between the least-square planes defined by the S1, S2, S3, and S4 atoms (Figs. 1 &2). Parallel π-stacks are interconnected with each other along the *b* axis by C1—H1···O1^i^ and C3—H3···O1^i^ (symmetry code: (i) -*x*, *y* + 1/2, -*z* + 1/2) short contacts, which can be classified as non-classical hydrogen bonds. Additionally, two S···S short contacts, which may be similar in nature to halogen bonds (Metrangolo *et al.*, 2008), are observed in the crystal structure. The shorter S2···S4^iv^ (3.4813 (7) Å) contacts (symmetry code: (iv) *x*, 1 + *y*, *z*) are observed along the *b* axis. The longer (3.4980 (9) Å) S3···S3^ii/iii^ contacts (symmetry codes: (ii) -1/2 + *x*, 1/2 - *y*, -*z*; (iii) 1/2 + *x*, 1/2 - *y*, -*z*) bind parallel π-stacks with each other along the *c* axis.

### Experimental

The title compound was prepared as described by Garín *et al.* (1994) by treatment of monolithio-TTF with *N*-methyl-*N*-phenylformamide in dry Et_2_O. The product was obtained as a deep red microcrystalline solid. Crystals suitable for X-ray diffraction were grown by slow evaporation of a solution in benzene/cyclohexane. Mp: 382–383 K; Lit: 382–383 K (Garín *et al.*, 1994). ^1^H NMR (200 MHz, CDCl_3_): δ 6.33 (d, *J* = 6.5 Hz, 1 H), 6.36 (d, *J* = 6.2 Hz, 1 H), 7.42 (s, 1 H), 9.48 (s, 1 H).

### Refinement

Hydrogen atoms were included at calculated positions using a riding model with aromatic and formyl C—H = 0.95. The *U*_iso_(H) values were fixed at 1.2 × *U*_eq_(C) of the parent C atom.

### Crystal data

**Table:**

C_7_H_4_OS_4_	*D*_x_ = 1.772 Mg m^−^^3^
*M**_r_* = 232.34	Melting point: 383 K
Orthorhombic, *P*2_1_2_1_2_1_	Mo *K*α radiation, λ = 0.71073 Å
*a* = 3.8466 (3) Å	Cell parameters from 4789 reflections
*b* = 7.4052 (7) Å	θ = 2.8–31.4°
*c* = 30.577 (3) Å	µ = 1.03 mm^−^^1^
*V* = 870.99 (13) Å^3^	*T* = 100 K
*Z* = 4	Plate, red
*F*(000) = 472	0.50 × 0.21 × 0.13 mm

### Data collection

**Table:**

Bruker SMART APEX CCD diffractometer	2663 reflections with *I* > 2σ(*I*)
Radiation source: fine focus sealed tube	*R*_int_ = 0.015
ω and φ scans	θ_max_ = 31.9°, θ_min_ = 1.3°
Absorption correction: multi-scan (*SADABS* in *APEX2*; Bruker, 2012)	*h* = −5→5
*T*_min_ = 0.675, *T*_max_ = 0.746	*k* = −10→10
6998 measured reflections	*l* = −42→44
2734 independent reflections	

### Refinement

**Table:**

Refinement on *F*^2^	Secondary atom site location: difference Fourier map
Least-squares matrix: full	Hydrogen site location: inferred from neighbouring sites
*R*[*F*^2^ > 2σ(*F*^2^)] = 0.024	H-atom parameters constrained
*wR*(*F*^2^) = 0.057	*w* = 1/[σ^2^(*F*_o_^2^) + (0.0237*P*)^2^ + 0.4121*P*] where *P* = (*F*_o_^2^ + 2*F*_c_^2^)/3
*S* = 1.13	(Δ/σ)_max_ < 0.001
2734 reflections	Δρ_max_ = 0.45 e Å^−^^3^
109 parameters	Δρ_min_ = −0.25 e Å^−^^3^
0 restraints	Absolute structure: Flack x determined using 985 quotients [(I+)-(I-)]/[(I+)+(I-)] (Parsons & Flack, 2004), 1024 Friedel pairs.
Primary atom site location: structure-invariant direct methods	Flack parameter: 0.01 (4)

### Special details

**Table:**

Geometry. All e.s.d.'s (except the e.s.d. in the dihedral angle between two l.s. planes) are estimated using the full covariance matrix. The cell e.s.d.'s are taken into account individually in the estimation of e.s.d.'s in distances, angles and torsion angles; correlations between e.s.d.'s in cell parameters are only used when they are defined by crystal symmetry. An approximate (isotropic) treatment of cell e.s.d.'s is used for estimating e.s.d.'s involving l.s. planes.

### Fractional atomic coordinates and isotropic or equivalent isotropic displacement parameters (Å^2^)

**Table:**

	*x*	*y*	*z*	*U*_iso_*/*U*_eq_	
C1	0.1960 (6)	0.3626 (3)	0.24133 (7)	0.0229 (4)	
H1	0.0987	0.4745	0.2504	0.027*	
C2	0.3148 (6)	0.3446 (3)	0.19636 (7)	0.0178 (4)	
C3	0.3234 (6)	0.4823 (3)	0.16743 (7)	0.0188 (4)	
H3	0.2417	0.5992	0.1751	0.023*	
C4	0.5823 (5)	0.2086 (3)	0.12667 (7)	0.0154 (4)	
C5	0.7332 (5)	0.1004 (3)	0.09665 (7)	0.0154 (4)	
C6	0.9971 (6)	−0.1700 (3)	0.05523 (7)	0.0213 (4)	
H6	1.0784	−0.2866	0.0473	0.026*	
C7	1.0078 (7)	−0.0331 (3)	0.02682 (7)	0.0213 (4)	
H7	1.0959	−0.0492	−0.0019	0.026*	
O1	0.2158 (5)	0.2405 (2)	0.26790 (5)	0.0275 (4)	
S1	0.47171 (14)	0.13395 (6)	0.17941 (2)	0.01732 (10)	
S2	0.48840 (15)	0.43791 (6)	0.11609 (2)	0.01750 (10)	
S3	0.85438 (14)	0.17653 (7)	0.04434 (2)	0.01774 (11)	
S4	0.82962 (14)	−0.12822 (7)	0.10718 (2)	0.01786 (11)	

### Atomic displacement parameters (Å^2^)

**Table:**

	*U*^11^	*U*^22^	*U*^33^	*U*^12^	*U*^13^	*U*^23^
C1	0.0258 (11)	0.0227 (9)	0.0202 (10)	0.0009 (9)	−0.0027 (8)	−0.0048 (8)
C2	0.0168 (9)	0.0174 (9)	0.0190 (9)	0.0009 (8)	−0.0026 (7)	−0.0033 (7)
C3	0.0204 (10)	0.0154 (8)	0.0206 (10)	0.0022 (8)	−0.0014 (9)	−0.0033 (7)
C4	0.0151 (9)	0.0134 (8)	0.0177 (9)	−0.0006 (6)	−0.0023 (7)	0.0024 (6)
C5	0.0150 (9)	0.0132 (8)	0.0180 (9)	−0.0011 (6)	−0.0020 (7)	0.0027 (7)
C6	0.0193 (9)	0.0186 (9)	0.0260 (10)	0.0023 (9)	−0.0008 (9)	−0.0069 (7)
C7	0.0201 (10)	0.0217 (9)	0.0221 (10)	0.0006 (9)	0.0005 (9)	−0.0056 (7)
O1	0.0359 (10)	0.0274 (8)	0.0192 (7)	−0.0016 (8)	−0.0012 (7)	−0.0002 (6)
S1	0.0208 (2)	0.01368 (19)	0.0175 (2)	0.00034 (19)	0.00125 (19)	0.00193 (17)
S2	0.0205 (2)	0.01230 (18)	0.0197 (2)	0.00120 (19)	−0.0006 (2)	0.00242 (16)
S3	0.0183 (2)	0.0181 (2)	0.0169 (2)	0.00035 (19)	0.00033 (19)	0.00149 (17)
S4	0.0192 (2)	0.01267 (19)	0.0217 (2)	0.00182 (19)	−0.00120 (19)	0.00094 (17)

### Geometric parameters (Å, º)

**Table:**

C1—O1	1.218 (3)	C4—S2	1.7661 (19)
C1—C2	1.455 (3)	C5—S3	1.759 (2)
C1—H1	0.9500	C5—S4	1.763 (2)
C2—C3	1.350 (3)	C6—C7	1.336 (3)
C2—S1	1.751 (2)	C6—S4	1.742 (2)
C3—S2	1.725 (2)	C6—H6	0.9500
C3—H3	0.9500	C7—S3	1.745 (2)
C4—C5	1.349 (3)	C7—H7	0.9500
C4—S1	1.757 (2)		
			
O1—C1—C2	122.9 (2)	C4—C5—S4	122.44 (15)
O1—C1—H1	118.6	S3—C5—S4	114.71 (12)
C2—C1—H1	118.6	C7—C6—S4	118.03 (16)
C3—C2—C1	123.9 (2)	C7—C6—H6	121.0
C3—C2—S1	118.05 (16)	S4—C6—H6	121.0
C1—C2—S1	118.02 (16)	C6—C7—S3	117.70 (17)
C2—C3—S2	117.48 (16)	C6—C7—H7	121.1
C2—C3—H3	121.3	S3—C7—H7	121.1
S2—C3—H3	121.3	C2—S1—C4	94.30 (10)
C5—C4—S1	122.80 (15)	C3—S2—C4	95.26 (10)
C5—C4—S2	122.29 (15)	C7—S3—C5	94.82 (10)
S1—C4—S2	114.90 (11)	C6—S4—C5	94.69 (10)
C4—C5—S3	122.85 (15)		
			
O1—C1—C2—C3	−174.0 (2)	C5—C4—S1—C2	178.23 (18)
O1—C1—C2—S1	3.4 (3)	S2—C4—S1—C2	−0.66 (13)
C1—C2—C3—S2	177.51 (17)	C2—C3—S2—C4	−0.5 (2)
S1—C2—C3—S2	0.1 (3)	C5—C4—S2—C3	−178.19 (18)
S1—C4—C5—S3	−178.29 (12)	S1—C4—S2—C3	0.71 (13)
S2—C4—C5—S3	0.5 (3)	C6—C7—S3—C5	−1.6 (2)
S1—C4—C5—S4	0.8 (3)	C4—C5—S3—C7	−178.51 (18)
S2—C4—C5—S4	179.63 (12)	S4—C5—S3—C7	2.32 (14)
S4—C6—C7—S3	0.3 (3)	C7—C6—S4—C5	1.2 (2)
C3—C2—S1—C4	0.3 (2)	C4—C5—S4—C6	178.61 (18)
C1—C2—S1—C4	−177.22 (18)	S3—C5—S4—C6	−2.21 (14)

### Hydrogen-bond geometry (Å, º)

**Table:**

*D*—H···*A*	*D*—H	H···*A*	*D*···*A*	*D*—H···*A*
C1—H1···O1^i^	0.95	2.38	3.228 (3)	149
C3—H3···O1^i^	0.95	2.69	3.445 (3)	137

Symmetry code: (i) −*x*, *y*+1/2, −*z*+1/2.

### Sulfur-sulfur short contacts (Å) of the title compound

**Table:**

System S···S	S···S	Symmetry code
S3···S3^ii^	3.4980 (9)	(ii) -1/2+x, 1/2-y, -z
S3···S3^iii^	3.4980 (9)	(iii) 1/2+x, 1/2-y, -z
S2···S4^iv^	3.4813 (7)	(iv) x, 1+y, z
